# Aid the Red-Cross Organizations

**Published:** 1898-08

**Authors:** 


### AID THE RED-CROSS ORGANIZATIONS.

No organization is better known throughout our broad land and in many foreign countries than the "Order of the Red Cross." Where it goes it carries gladness, comfort, assistance, and every solace, sympathy, and aid that tend to lessen every form and kind of suffering in every nook and corner of the world. In the spreading of its wings of peace and good cheer ; in the open hand of assistance, ever ready to lend substantial aid, with its self-sacrificing women and men identified with it for these blessed purposes, in all its ramifications, it knows no nationality, creed or rank ; but to one and all tenders and pours forth its kindness and hospitable attentions and support. It needs at home the recognition, support, and practical aid of every good citizen of our land. It needs yours, fellow-veterinarians. It is worthy of every support and aid you can give it. See to it that the branch in your village, town, or city, county or State receives your aid and assistance, and join in the feeling that the world is growing better in that the fellowship of man grows stronger and closer.

